# Studies on the Synthesis of Derivatives of Marine-Derived Bostrycin and Their Structure-Activity Relationship against Tumor Cells

**DOI:** 10.3390/md10040932

**Published:** 2012-04-24

**Authors:** Hong Chen, Lili Zhong, Yuhua Long, Jia Li, Jueheng Wu, Lan Liu, Shengping Chen, Yongcheng Lin, Mengfeng Li, Xun Zhu, Zhigang She

**Affiliations:** 1 School of Chemistry and Chemical Engineering, Sun Yat-sen University, 135 Xingang West Road, Guangzhou 510275, China; Email: chenwexpo@sina.com (H.C.); zhonglili42@yahoo.com.cn (L.Z.); nuekagami@163.com (J.L.); cesllan@mail.sysu.edu.cn (L.L.); ceslyc@mail.sysu.edu.cn (Y.L.); 2 Guangdong Province Key Laboratory of Functional Molecules in Oceanic Microorganism, Bureau of Education, Sun Yat-sen University, 74 Zhongshan Road II, Guangzhou 510080, China; Email: wujh@mail.sysu.edu.cn (J.W.); chenshp@mail.sysu.edu.cn (S.C.); limf@mail.sysu.edu.cn (M.L.); 3 School of Chemistry and Environment, South China Normal University, 348 West Outer Ring Road, Guangzhou 510006, China; Email: longyh@scnu.edu.cn; 4 Department of Microbiology, Zhongshan School of Medicine, Sun Yat-sen University, 74 Zhongshan Road II, Guangzhou 510080, China; 5 Key Laboratory of Tropical Disease Control, Ministry of Education, Sun Yat-sen University, 74 Zhongshan Road II, Guangzhou 510080, China

**Keywords:** bostrycin, synthesis, antitumor, derivatives, structure-activity relationship

## Abstract

A series of new derivatives (**5**–**29**) of marine-derived bostrycin (**1**) were synthesized. The *in vitro* cytotoxic activities of all compounds were evaluated against MCF-7, MDA-MB-435, A549, HepG2, HCT-116 and MCF-10A cells using the MTT method. The compounds **7**, **8**, **22**, **23**, **25**, **28** and **29** of the total showed comparable activity to epirubicin, the positive control, against the tested cancer cell lines. However, these compounds also exhibited cytotoxicity towards MCF-10A cells. The structure-activity relationship (SAR) of bostrycin derivatives was also discussed based on the obtained experimental data.

## 1. Introduction

**Figure 1:**
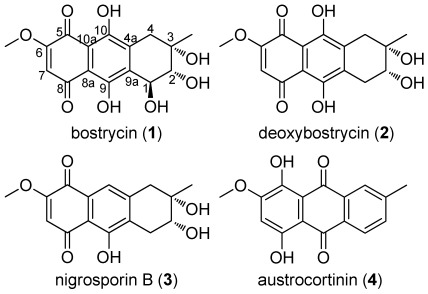
Structures of bostrycin (**1**), deoxybostrycin (**2**), nigrosporin B (**3**) and austrocortinin (**4**).

**Scheme 1:**
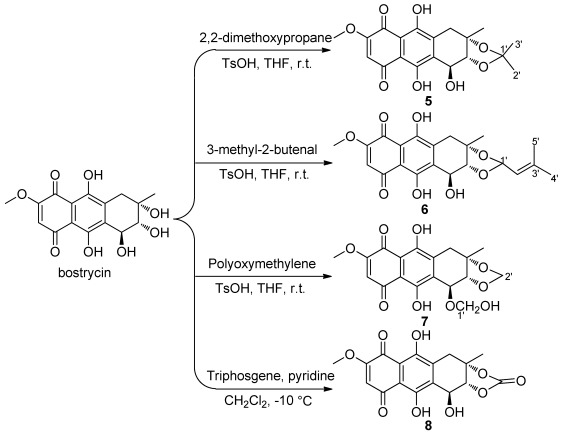
Synthesis of 2,3-ketal/substituted bostrycin derivatives **5**–**8**.

A variety of tetrahydroanthraquinone derivatives isolated from fungi [1,2,3,4,5,6] and plants [7] display various biological properties including antibacterial [3,6], antiprotozoal [8], phytotoxic [9] and cytotoxic activities [7,10]. Previous studies have shown that tetrahydroanthraquinone derivatives can inhibit the growth of cultured cells of *Nicotiana rustica*, and act as a potent stimulator of NADH oxidation in mitochondria and electron acceptors in an enzyme preparation of diaphorase [11]. Bostrycin (**1**, Figure 1), a natural tetrahydroanthraquinone compound, was isolated from the mangrove endophytic fungus No. 1403 collected from the South China Sea, as well as three bostrycin analogues (**2**–**4**, Figure 1) [12,13,14]. The originally proposed structure of bostrycin was identified by interpretation of spectral date (IR, UV, MS, ^1^H NMR, ^13^C NMR) [15,16,17], and then revised by Kelly and his co-workers [18] on the basis of the total synthesis of (+/−)-bostrycin and an X-ray crystal structure of the *O*-isopropylidene derivative. Subsequently, Larsen’s group [19] revised the absolute configuration of (−)-bostrycin through an elegant asymmetric synthesis of (+)-bostrycin. Our recent studies have shown that bostrycin has broad-spectrum antitumor activity [13,14,20,21]. Bostrycin can induce apoptosis of breast cancer cells through Akt/FOXO pathway, revealing it is a potent apoptosis inducer [20]. Bostrycin also showed lethal cytotoxicity to yeast cells by inducing apoptosis via an Aif1p-dependent, mitochondria-mediated apoptotic pathway [22]. Chen *et al*. [23] reported that bostrycin can inhibit proliferation of human lung carcinoma A549 cells via downregulation of the P13K/Akt. The pharmacological and toxicological profiles of bostrycin, as well as its *in vivo* antitumor efficacy, provided us with reasonable optimism that bostrycin could become a promising Akt inhibitor and anticancer drug candidate [20]. However, there have been few studies on the structural modification and structure-activity relationship (SAR) of bostrycin. Therefore, our research interest was focused on the modification of bostrycin and its SAR study with the intention of discovering novel antitumor agents from bostrycin derivatives. In this work, a series of bostrycin derivatives (**5**–**29**, Scheme 1–Scheme 3) were synthesized with variation at positions 2, 3, 6 and 7 of the tetrahydroanthraquinone core. All these natural and synthesized compounds were evaluated for their cytotoxic activities against five human cancer cell lines MCF-7, MDA-MB-435, A549, HepG2 and HCT-116, and one immortalized human breast epithelial cell line MCF-10A. The SAR was further discussed on the basis of the obtained experimental data. As we expected, some of the modified compounds exhibited strong anticancer activities against the tested cancer cells, with superior potency over the parent bostrycin.

**Scheme 2:**
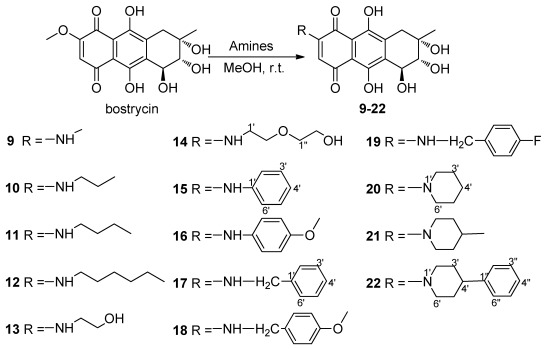
Synthesis of 6-aminosubstituted bostrycin derivatives **9**–**22**.

**Scheme 3:**
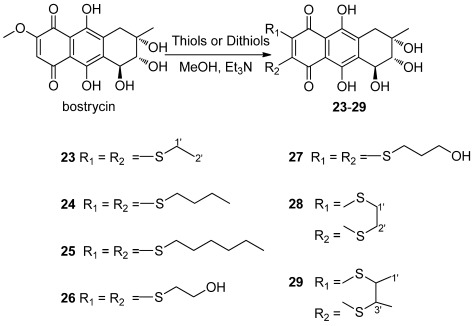
Synthesis of 6,7-thiosubstituted bostrycin derivatives **23**–**29**.

## 2. Results and Discussion

### 2.1. Chemistry

Bostrycin (**1**) and its analogues (**2**–**4**, Figure 1) were isolated from the mangrove endophytic fungus No. 1403 collected from the South China Sea [12,13,14]. To explore the effects of hydroxyl groups at C-2 and C-3 positions in bostrycin on antitumor activity, 2,3-ketal/substituted derivatives **5**–**8** were synthesized (Scheme 1). Bostrycin was reacted with 2,2-dimethoxypropane, 3-methyl-2-butenal and polyoxymethylene in the presence of 1 equiv of *p*-toluenesulfonic acid (TsOH) at room temperature, 2,3-ketal derivatives **5**, **6** and **7** were obtained respectively. Moreover, treatment of Bostrycin with triphosgene in the presence of pyridine at −10 °C gave compound **8**.

**Figure 2:**
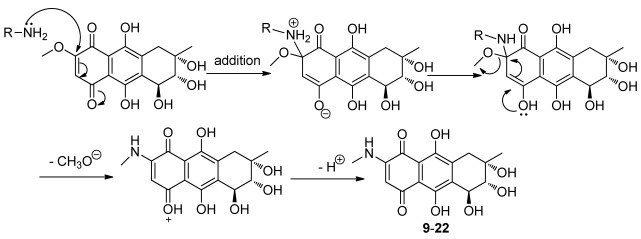
Nucleophilic substitution of bostrycin and amines.

**Figure 3:**
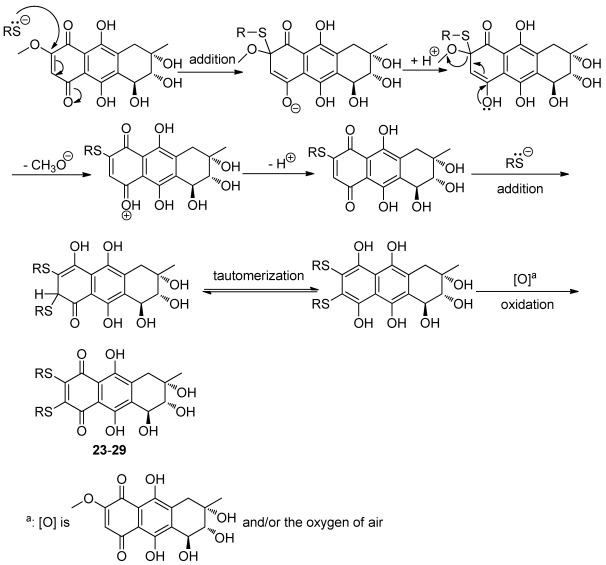
Nucleophilic substitution of bostrycin and thiols.

The nucleophilic substitution of 2,3-dichloro-1,4-naphthoquinone by various nucleophiles was employed as the key reaction [24,25,26,27] to afford the structurally diverse derivatives with variation on the C-6 and C-7 positions of bostrycin. We studied the reaction of bostrycin with various amines (Scheme 2) and thiols/dithiols (Scheme 3). When bostrycin was treated with various types of amines at room temperature or 50 °C using methanol as solvent, a series of alkyl/arylamino derivatives **9**–**22** were obtained (Scheme 2). In addition, the preparation of compounds **15** and **16** required a longer reaction time and higher temperature presumably due to the low reactivity of the aryl amines used. This reaction involved a typical nucleophilic displacement of the methoxy group in bostrycin (**1**) with various amines as nucleophiles. The possible mechanism is depicted as in Figure 2. When bostrycin reacted with various thiols and dithiols at 0-5 °C in the presence of triethylamine, a series of alkylthio disubstituted derivatives **23**–**29** (at C-6 and C-7) were obtained (Scheme 3). We deduced the dithio substituted compounds might be obtained through two steps. Firstly, nucleophilic addition of an alkylthio anion to C-6 followed by elimination of the methoxy group to give a monothio substituted bostrycin intermediate. However, the second addition of another mole of alkylthio anion to C-7 can be smoothly carried out due to its strong nucleophilicity, and then tautomerization between the ketone and enol configuration takes place. Subsequently, the diphenol structure was oxidized to the target quinone structure (**23**–**29**) by the remaining large amount of starting material bostrycin and/or oxygen from the air. The detailed mechanism is proposed as in Figure 3. The structures of all the compounds were established on the basis of their nuclear magnetic resonance spectra (^1^H NMR, ^13^C NMR) and mass spectra (ESI, EI).

### 2.2. Biological Activity

All compounds were evaluated for their *in vitro* cytotoxic activity against five human cancer cell lines including human breast MCF-7, human breast MDA-MB-435, human lung A549, human liver HepG2 and human colon HCT-116, and compared with their effects on the immortalized human breast epithelial cell line MCF-10A by MTT assay [20,28] using epirubicin (an anticancer drug used widely in the clinic [29,30,31]) as positive control. The results are summarized in Table 1 and discussed below.

**Table 1:** Cytotoxicity of compounds **1**–**29 **against MCF-7, MDA-MB-435, A549, HepG2, HCT-116 and MCF-10Acells (IC_50_, μM)^a^.

Compound/Cell Line	MCF-7 ^b^	MDA-MB-435 ^b^	A549 ^b^	HepG2 ^b^	HCT-116 ^b^	MCF-10A ^b^
**1**	2.18 ± 0.14	2.82 ± 0.17	2.63 ± 0.33	7.71 ± 0.72	4.78 ± 0.03	14.08 ± 0.58
**2**	2.69 ± 0.31	3.19 ± 0.92	4.49 ± 0.13	9.99 ± 0.55	5.69 ± 0.25	13.83 ± 0.76
**3**	6.19 ± 0.60	2.91 ± 0.18	4.72 ± 0.17	31.01 ± 0.71	4.70 ± 0.39	28.63 ± 0.91
**4**	>50	41.59 ± 2.63	>50	>50	>50	>50
**5**	6.87 ± 0.33	5.96 ± 0.21	4.25 ± 0.16	36.95 ± 1.97	11.03 ± 1.35	18.11±1.30
**6**	7.99 ± 0.09	11.67 ± 1.18	5.50 ± 0.24	22.34 ± 2.89	7.83 ± 0.32	34.15 ± 0.85
**7**	2.52 ± 0.26	4.25 ± 0.57	0.78 ± 0.04	4.58 ± 0.50	3.06 ± 0.10	4.79 ± 0.52
**8**	1.59 ± 0.16	1.26 ± 0.12	0.52 ± 0.02	3.32 ± 0.58	2.92 ± 0.06	21.72 ± 1.45
**9**	3.58 ± 0.65	>50	31.07 ± 1.94	22.35 ± 1.16	34.24 ± 1.27	>50
**10**	13.97 ± 0.98	>50	21.50 ± 1.43	18.00 ± 0.60	26.38 ± 0.89	>50
**11**	1.08 ± 0.21	>50	25.14 ± 0.74	35.68 ± 1.29	36.46 ± 0.53	>50
**12**	4.87 ± 0.23	4.25 ± 0.75	3.74 ± 0.67	12.91 ± 0.96	4.06 ± 0.65	Nt ^c^
**13**	35.55 ± 1.40	>50	>50	32.16 ± 2.43	35.93 ± 1.13	>50
**14**	>50	>50	>50	>50	>50	>50
**15**	3.72 ± 0.27	10.32 ± 0.45	4.87 ± 0.43	>50	4.84 ± 0.25	41.60 ± 0.51
**16**	39.44 ± 0.68	36.41 ± 2.08	41.44 ± 2.86	30.31 ± 1.63	43.27 ± 1.56	Nt ^c^
**17**	28.66 ± 2.37	40.37 ± 1.49	21.30 ± 2.02	>50	20.63 ± 0.37	19.79 ± 0.63
**18**	9.17 ± 0.28	25.56 ± 1.71	15.18 ± 0.42	>50	13.63 ± 1.85	27.69 ± 0.93
**19**	7.94 ± 0.26	7.86 ± 1.20	6.25 ± 0.43	>50	4.92 ± 0.38	7.50 ± 0.69
**20**	3.14 ± 0.31	4.04 ± 0.35	3.16 ± 0.23	1.99 ± 0.17	2.81 ± 0.31	Nt ^c^
**21**	4.62 ± 0.44	6.38 ± 0.47	3.06 ± 0.46	6.09 ± 1.14	3.25 ± 0.67	Nt ^c^
**22**	4.81 ± 0.40	0.95 ± 0.13	0.76 ± 0.03	6.61 ± 1.12	0.75 ± 0.05	Nt ^c^
**23**	0.71 ± 0.01	0.76 ± 0.06	4.07 ± 0.51	6.90 ± 0.26	0.95 ± 0.06	14.79 ± 0.96
**24**	2.45 ± 0.48	7.05 ± 0.56	4.06 ± 0.42	4.03 ± 0.13	3.25 ± 0.54	30.64 ± 2.54
**25**	2.60 ± 0.45	10.64 ± 1.11	0.71 ± 0.01	3.33 ± 0.66	0.74 ± 0.10	34.96 ± 1.94
**26**	14.42 ± 0.42	13.33 ± 0.20	26.12 ± 3.15	22.66 ± 1.95	11.95 ± 1.53	Nt ^c^
**27**	6.01 ± 0.39	3.19 ± 0.15	7.31 ± 0.58	3.18 ± 0.75	3.08 ± 0.35	Nt ^c^
**28**	0.57 ± 0.04	0.63 ± 0.45	0.37 ± 0.04	0.82 ± 0.01	0.68 ± 0.08	0.81 ± 0.11
**29**	3.18 ± 0.22	0.55 ± 0.11	4.06 ± 0.35	2.55 ± 0.55	0.73 ± 0.02	Nt ^c^
epirubicin ^d^	0.96 ± 0.08	0.56 ± 0.06	0.61 ± 0.05	0.96 ± 0.02	0.48 ± 0.03	0.48 ± 0.08

^a^ IC_50_ values are taken as means ± standard deviation from three independent experiments; ^b^ MCF-7, human breast cancer cell line; MDA-MB-435, human breast cancer cell line; A549, human lung cancer cell line; HepG2, human liver cancer cell line; HCT-116, human colon cancer cell line; MCF-10A, the immortalized human breast epithelial cell line; ^c ^Nt = not tested; ^d^ Used as a positive control.

Bostrycin analogues **2** and **3 **exhibited comparable cytotoxic activity to bostrycin against all tested cancer cell lines, except for compound **3 **for HepG2 cells, while compound **4** lost potency (IC_50_ > 50 μM). The activity profiles suggested that the hydroxyl groups at C-1 and/or C-10 in bostrycin were not essential for the cytotoxic activity whereas the tetrahydroaromatic ring with the polyhydroxyl groups was the key pharmacophore of bostrycin.

These bostrycin derivatives were tested on inhibitory activity against the growth of several tumor cell lines along with one immortalized human breast epithelial cell line. As shown in Table 1, some modified compounds exhibited much better activity than bostrycin, and even displayed comparable activity to epirubicin against the cancer cells tested. However, the majority of compounds were also highly cytotoxic to the immortalized human breast epithelial cells. For example, the activities of compounds **7** (IC_50_ = 0.78 μM) and **8 **(IC_50_ = 0.52 μM) against A549 cells have comparable activity to those of epirubicin (IC_50_ = 0.61 μM). A similar potency profile was observed with compounds **22 **(against MDA-MB-435, A549 and HCT-116 cells), **23 **(against MCF-7, MDA-MB-435 and HCT-116 cells), **25** (against A549 and HCT-116 cells) and **29 **(against MDA-MB-435 and HCT-116 cells). Significantly, compound **28** showed broad-spectrum antitumor activity, with equal potency to epirubicin against MCF-7, MDA-MB-435, A549, HepG2 and HCT-116 cells lines with IC_50_ values of 0.57, 0.63, 0.37, 0.82 and 0.68 μM, which were 3.8-, 4.5-, 7.1-, 9.4- and 7.0-fold more active than bostrycin (with IC_50_ value of 2.18, 2.82, 2.63, 7.71 and 4.78 μM), respectively. However, these compounds also exerted marked cytotoxic effect on immortalized human breast epithelial cells. Moreover, compounds **7** and **8 **exhibited excellent selective activity for A549 cells over other cancer cells, and the cytotoxic activity of compounds **9** and **11 **against MCF-7 cell line was much stronger than for other kinds of cancer cell lines. Compound **28 **(IC_50_ = 0.57 μM) possessed the most potent activity against HepG2 cells. The SAR analysis revealed that: (1) 2,3-ketal derivatives **5** and **6** showed a slight decrease in antiproliferative activity compared to bostrycin. However, compound **8 **displayed stronger activity, which indicated that the protection of C-2 and C-3 with a dioxylcarbonyl group was favorable for activity; (2) Compound **7** with a hydroxymethyl group at C-1 position and a methylene group at C-2 and C-3 positions, showed comparable activity to epirubicin for A549 cells; (3) Secondary heterocyclic amine-substituted derivatives (**20**–**22**) possessed more potency than primary amine-substituted derivatives (**9**–**11 **and **13**–**19**) against all cancer cells except for MCF-7 cells. The results suggested that derivatives bearing tertiary amino groups have better activity than derivatives bearing secondary amino groups; (4) Although compounds **9** and **11 **displayed significantly decreased potency compared to that of bostrycin against five human cancer cells except for MCF-7 cells, they had improved selectivity for MCF-7 cells over other cancer cells; (5) Compared to alkylamino derivatives **9**–**11**, longer alkylamino substituents seems beneficial for anticancer activity, as exemplified by compound **12 **with significantly improved activity against MDA-MB-435, A549 and HCT-116 cells; (6) Phenylamino-substituted derivative **15** exhibited better antiproliferative activity than all other primary amine-substituted derivatives against A549 and HCT-116 cells, except for compound **12**; (7) Compared to derivatives **20**, **21**, 4-phenylpiperidine-substituted derivative **22** exhibited comparable activity to epirubicin against MDA-MB-435, A549 and HCT-116 cells, which indicated that a larger group substituted at the 6-piperidine position may be helpful for activity; (8) Disubstituted thiol derivatives **23**–**25 **and **27** showed strong cytotoxic activity against five human cancer cell lines with IC_50_ values of 0.71-10.64 μM; (9) The length of the carbon chain of alkylthio substituents also affected the antiproliferative activity of the compounds against different cancer cells. For example, for compounds **23**–**25**, the activity to inhibit the growth of MCF-7 and MDA-MB-435 cells was decreased as the length of the carbon chain of alkylthio substituents was increased. However, for A549 and HepG2 cells, the order of activity was reversed; (10) Dithiol-substituted derivatives **28**, **29 **displayed potent cytotoxic activity against five human cancer cell lines, especially, compound **28** which generally exhibited comparable activity to epirubicin against five human cancer cell lines.

## 3. Experimental Section

### 3.1. Chemistry

Reagents and solvents were commercially available. Solvents were dried and purified using standard procedures prior to use. Melting points were measured on an X-4 micromelting point apparatus and were uncorrected. IR spectra were measured on a Bruker Vector 22 spectrophotometer using KBr pellets. NMR spectra were determined on a Varian Inova-500 NB spectrometer or Bruker AV-400 NB spectrometer in CDCl_3_ or DMSO-*d*_6_ using TMS as internal standard, and coupling constants (*J*) are in Hz. EI mass spectra were recorded on a DSQ mass spectrometer and ESI mass spectra were obtained on a LCQ DECA XP LC-MS mass spectrometer. Flash column chromatography was performed with silica gel (Qing dao Ocean Chemical Factory, 200–300 mesh) eluted with petroleum ether–dichloromethane or dichloromethane–methanol, and C18 reversed phase silica gel (Welch Material, Inc., 45 μm) eluted with methanol–water.

### 3.2. Preparation of Bostrycin ***(1)***, Deoxybostrycin ***(2)***, Nigrosporin B ***(3)*** and Austrocortinin ***(4)***

Bostrycin (**1**) and its analogues (**2**–**4**) were isolated from the secondary metabolites of the mangrove endophytic fungus *Nigrospora* sp. No. 1403 collected from the South China Sea according to literature procedures [12,13,14].

Bostrycin (**1**). Red solid (MeOH); mp: 252–254 °C; [α]^25^_D_ −272 (*c* 2.2 × 10^−4^, MeOH); IR (KBr): ν_max_ = 3532, 3512, 3480, 3367, 3031, 2991, 2934, 2893, 2857, 1595, 1478, 1460, 1441 cm^−1^; ^1^H NMR (400 MHz, DMSO-*d*_6_): δ 13.39 (s, 1H, 9-OH), 12.63 (s, 1H, 10-OH), 6.48 (s, 1H, 7-H), 5.23 (d, *J* = 4.9 Hz, 1H, 1-OH), 4.93 (d, *J* = 4.8 Hz, 1H, 2-OH), 4.75 (t, *J *= 4.9 Hz, 1H, 1-H), 4.48 (s, 1H, 3-OH), 3.92 (s, 3H, 6-OMe), 3.53 (t, *J *= 4.8 Hz, 1H, 2-H), 2.74 (d, *J *= 18.2 Hz, 1H, 4-H_b_), 2.67 (d, *J *= 18.2 Hz, 1H, 4-H_a_), 1.23 (s, 3H, 3-CH_3_); ^13^C NMR (125 MHz, DMSO-*d*_6_): δ 184.29 (C-8), 177.49 (C-5), 160.45 (C-9), 160.22 (C-6), 160.07 (C-10), 139.44 (C-9a), 136.80 (C-4a), 109.85 and 109.51 (C-7 and C-10a), 107.49 (C-8a), 76.40 (C-2), 69.33 (C-3), 68.30 (C-1), 56.97 (6-OMe), 34.89 (C-4), 25.67 (3-CH_3_); EIMS *m/z* 336 [M]^+^ (23), 318 (17), 303 (9), 289 (19), 271 (12), 263 (100), 247 (11), 234 (35), 216 (16).

Deoxybostrycin (**2**). Red solid (MeOH); mp: 224–225 °C; [α]^25^_D_ +90.9 (c 1.1 × 10^−4^, MeOH); ^1^H NMR (300 MHz, DMSO-*d*_6_): δ 13.18 (s, 1H, 9-OH), 12.62 (s, 1H, 10-OH), 6.42 (s, 1H, 7-H), 4.82 (d, *J *= 5.1 Hz, 1H, 2-OH), 4.48 (s, 1H, 3-OH), 3.88 (s, 3H, 6-OMe), 3.61 (dt, *J *= 7.3, 5.1 Hz, 1H, 2-H), 2.82 (dd, *J *= 18.3, 5.1 Hz, 1H, 1-H_b_), 2.77 (d, *J *= 18.1 Hz, 1H, 4-H_b_), 2.64 (dd, *J *= 18.3, 7.3 Hz, 1H, 1-H_a_), 2.56 (d, *J *= 18.1 Hz, 1H, 4-H_a_), 1.18 (s, 3H, 3-CH_3_); ^13^C NMR (125 MHz, DMSO-*d*_6_): δ 183.39 (C-8), 176.35 (C-5), 161.31 (C-10), 160.38 (C-6), 159.67 (C-9), 138.88 (C-9a), 136.33 (C-4a), 109.47 (C-7), 108.97 (C-10a), 106.85 (C-8a), 70.12 (C-2), 68.81 (C-3), 56.95 (6-OMe), 35.72 (C-4), 29.91 (C-1), 25.39 (3-CH_3_); EIMS *m/z* 320 [M]^+^ (100), 302 (41), 287 (30), 259 (61), 247 (96), 234 (20), 219 (45), 205 (10).

Nigrosporin B (**3**). Yellow solid (MeOH); mp: >300 °C; [α]^25^_D_ +131.4 (c 1.75 × 10^−4^, MeOH); ^1^H NMR (400 MHz, DMSO-*d*_6_): δ 12.67 (s, 1H, 9-OH), 7.26 (s, 1H, 10-H), 6.29 (s, 1H, 7-H), 4.76 (d, *J *= 3.6 Hz, 1H, 2-OH), 4.39 (s, 1H, 3-OH), 3.88 (s, 3H, 6-OMe), 3.66 (m, 1H, 2-H), 2.93 (d, *J *= 17.5 Hz, 1H, 4-H_b_), 2.85 (dd, *J *= 18.4, 4.9 Hz, 1H, 1-H_b_), 2.77 (d, *J *= 17.5 Hz, 1H, 4-H_a_), 2.69 (dd, *J *= 18.4, 7.1 Hz, 1H, 4-H_a_), 1.17 (s, 3H, 3-CH_3_); ^13^C NMR (100 MHz, DMSO-*d*_6_): δ 190.76 (C-8), 178.79 (C-5), 161.11 (C-6), 158.31 (C-9), 144.01 (C-4a), 131.70 (C-9a), 127.95 (C-10a), 119.37 (C-10), 110.65 (C-8a), 109.33 (C-7), 70.51 (C-2), 69.41 (C-3), 56.78 (6-OMe), 41.67 (C-4), 29.75 (C-1), 25.07 (3-CH_3_); EIMS *m/z* 304 [M]^+^ (34), 286 (85), 271 (100), 257 (57), 243 (94), 229 (45), 215 (54), 201 (23).

Austrocortinin (**4**). Yellow solid (CH_2_Cl_2_); mp: 232–233 °C; ^1^H NMR (400 MHz, CDCl_3_): δ 13.59 (s, 1H, 8-OH), 13.47 (s, 1H, 5-OH), 8.25 (d, *J *= 8.0 Hz, 1H, 1-H), 8.16 (m, 1H, 4-H), 7.64 (d m, *J *= 8.0 Hz, 1H, 2-H), 6.72 (s, 1H, 7-H), 4.03 (s, 3H, 6-OMe), 2.57 (br s, 3H, 3-CH_3_); EIMS *m/z* 284 [M]^+^ (100), 266 (28), 238 (23), 210 (22), 182 (8), 157 (5), 139 (6).

### 3.3. Synthetic Methods of Compounds

#### 3.3.1. Synthesis of 2,3-*O*-(isopropylidene) Bostrycin **(5)**

To a solution of **1** (50 mg, 0.149 mmol) in 10 mL of tetrahydrofuran was added 2,2-dimethoxypropane (310 mg, 3.0 mmol) and *p*-toluenesulfonic acid (25.6 mg, 0.149 mmol). The reaction mixture was stirred for 20 h at room temperature. The reaction mixture was diluted with water (20 mL) and extracted with dichloromethane (3 × 50 mL). The combined organic phase was washed with saturated sodium chloride, dried over anhydrous magnesium sulfate, and concentrated *in vacuo*. The resulting residue was purified on a silica gel column using petroleum ether–dichloromethane (v/v 1:1) as eluent to obtain 48.5 mg of compound **5**. Yield 90%; red solid (CH_2_Cl_2_); mp: 228–229 °C; IR (KBr): ν_max_ = 3486, 3036, 2989, 2972, 2937, 2890, 1598, 1427, 1384 cm^−1^; ^1^H NMR (400 MHz, CDCl_3_) δ 13.18 (s, 1H, 9-OH), 12.60 (s, 1H, 10-OH), 6.18 (s, 1H, 7-H), 5.51 (d, *J *= 2.7 Hz, 1H, 1-H), 4.41 (d, *J *= 2.7 Hz, 1H, 2-H), 3.93 (s, 3H, 6-OMe), 3.41 (d, *J *= 16.7 Hz, 1H, 4-H_b_), 2.72 (d, *J *= 16.7 Hz, 1H, 4-H_a_), 1.64 (s, 3H, 3-CH_3_), 1.40 (s, 3H, 2′-CH_3_), 1.03 (s, 3H, 3′-CH_3_); EIMS *m/z* 376 [M]^+^ (19), 361 (8), 318 (83), 301 (33), 289 (100), 273 (43), 257 (24), 243 (16).

#### 3.3.2. Synthesis of 2,3-*O*-(3′-methylbut-2′-enyl) Bostrycin **(6)**

To a solution of **1** (50 mg, 0.149 mmol) in 10 mL of tetrahydrofuran was added 3-methyl-2-butenal (37.5 mg, 0.45 mmol) and *p*-toluenesulfonic acid (25.6 mg, 0.149 mmol). The reaction mixture was stirred for 10 h at room temperature. The reaction mixture was diluted with water (20 mL) and extracted with dichloromethane (3 × 50 mL). The combined organic phase was washed with saturated sodium chloride, dried over anhydrous magnesium sulfate, and concentrated *in vacuo*. The resulting residue was purified on a silica gel column using dichloromethane as eluent to obtain 47.9 mg of compound **6**. Yield 80%; red solid (CH_2_Cl_2_); mp: 200-201 °C; IR (KBr): ν_max_ = 3522, 3083, 2962, 2928, 2873, 1598, 1571, 1437, 1407, 1376 cm^−1^; ^1^H NMR (400 MHz, CDCl_3_) δ 13.09 (s, 1H, 9-OH), 12.53 (s, 1H, 10-OH), 6.12 (s, 1H, 7-H), 5.50 (d, *J *= 7.6 Hz, 1H, 1′-H), 5.45 (d, *J *= 2.5 Hz, 1H, 1-H), 4.72 (d septet, *J *= 7.6, 1.2 Hz, 1H, 2′-H), 4.20 (d, *J *= 2.5 Hz, 1H, 2-H), 3.92 (s, 3H, 6-OMe), 3.51 (d, *J *= 16.3 Hz, 1H, 4-H_b_), 2.67 (d, *J *= 16.3 Hz, 1H, 4-H_a_), 1.67 and 1.60 (d, *J *= 1.2 Hz, 4′-CH_3_ and 5′-CH_3_), 1.57 (s, 3H, 3-CH_3_); ^13^C NMR (100MHz, CDCl_3_): δ 186.45 (C-8), 180.60 (C-5), 160.69 (C-6), 158.54 (C-10), 156.95 (C-9), 141.85 (C-3′), 138.10 (C-9a), 137.86 (C-4a), 120.51 (C-2′), 110.97 (C-10a), 109.92 (C-7), 108.79 (C-8a), 97.27 (C-1′), 83.05 (C-2), 79.12 (C-3), 62.82 (C-1), 56.77 (6-OMe), 31.27 (C-4), 26.34 (3-CH_3_), 25.73 and 18.39 (C-4′ and C-5′); EIMS *m/z* 402 [M]^+^ (5), 387 (2), 318 (100), 301 (42), 289 (96), 271 (39), 257 (26), 243 (19).

#### 3.3.3. Synthesis of 1-*O*-(hydroxymethyl)-2,3-*O*-(methylene) Bostrycin **(7)**

To a solution of **1 **(100 mg, 0.298 mmol) in 10 mL of tetrahydrofuran was added polyoxymethylene (45 mg) and *p*-toluenesulfonic acid (51.2 mg, 0.298 mmol). The reaction mixture was stirred for 10 h at room temperature. The reaction mixture was diluted with water (20 mL) and extracted with dichloromethane (3 × 50 mL). The combined organic phase was washed with saturated sodium chloride, dried over anhydrous magnesium sulfate, and concentrated *in vacuo*. The resulting residue was purified on a silica gel column using dichloromethane–methanol (v/v 200:1) as eluent to obtain 27 mg of compound **7**. Yield 24%; red solid (CH_2_Cl_2_); mp: 233–234 °C; IR (KBr): ν_max_ = 3519, 3040, 2956, 2927, 2901, 2871, 1603, 1568, 1460, 1435, 1408 cm^−1^; ^ 1^H NMR (400 MHz, CDCl_3_): δ 13.23 (s, 1H, 9-OH), 12.64 (s, 1H, 10-OH), 6.17 (s, 1H, 7-H), 5.23 (d, *J *= 7.1 Hz, 1H, 1′-H_b_), 5.19 (d, *J *= 4.4 Hz, 1H, 2′-H_b_), 5.12 (dd, *J *= 7.5, 1.6 Hz, 1H, 2-H), 5.07 (d, *J *= 4.4 Hz, 1H, 2′-H_a_), 5.04 (d, *J *= 7.1 Hz, 1H, 1′-H_a_), 3.92 (s, 3H, 6-OMe), 3.63 (d, *J *= 7.5 Hz, 1H, 1-H), 3.18 (d, *J *= 18.9 Hz, 1H, 4-H_b_), 2.69 (dd, *J *= 18.9, 1.6 Hz, 1H, 4-H_a_), 1.50 (s, 3H, 3-CH_3_); ^13^C NMR (100 MHz, CDCl_3_): δ 185.53 (C-8), 179.56 (C-5), 160.47 (C-6), 159.42 and 159.38 (C-9 and C-10), 136.41 (C-9a), 135.98 (C-4a), 110.53 (C-10a), 110.12 (C-7), 108.64 (C-8a), 93.69 and 91.57 (C-1′ and C-2′), 84.98 (C-2), 75.52 (C-3), 70.64 (C-1), 56.71 (6-OMe), 36.47 (C-4), 26.25 (3-CH_3_); EIMS *m/z* 378 [M]^+^ (86), 348 (14), 330 (45), 300 (100), 272 (80), 257 (45), 229 (28), 203 (11).

#### 3.3.4. Synthesis of 2,3-*O*-(carbonyl) Bostrycin **(8)**

To a solution of **1 **(100 mg, 0.298 mmol) and pyridine (235 mg, 0.298 mmol) in 5 mL of dichloromethane at −10 °C was added a solution of triphosgene in 2 mL of dichloromethane dropwise. The reaction mixture was stirred for 30 min, and then a saturated aqueous solution of ammonium chloride (2 mL) was added. The reaction mixture was diluted with water (10 mL) and extracted with dichloromethane (3 × 50 mL). The combined organic phase was successively washed with dilute aqueous hydrochloric acid (2 M), water, saturated sodium chloride, dried over anhydrous magnesium sulfate, and concentrated *in vacuo*. The resulting residue was purified on a silica gel column using dichloromethane–methanol (v/v 200:1) as eluent to obtain 30.2 mg of compound **8**. Yield 28%; red solid (MeOH); mp: 246–247 °C; IR (KBr): ν_max_ = 3403, 3081, 2977, 2942, 2850, 1814, 1603, 1570, 1435, 1411 cm^−1^; ^1^H NMR (400 MHz, DMSO-*d*_6_): δ 13.14 (s, 1H, 9-OH), 12.32 (s, 1H, 10-OH), 6.45 (s, 1H, 7-H), 6.20 (d, *J *= 4.1 Hz, 1H, 1-OH), 5.33 (dd, *J *= 4.1, 2.9 Hz, 1H, 1-H), 4.88 (d, *J *= 2.9 Hz, 1H, 2-H), 3.92 (s, 3H, 6-OMe), 3.54 (d, *J *= 16.5 Hz, 1H, 4-H_b_), 2.81 (d, *J *= 16.5 Hz, 1H, 4-H_a_), 1.76 (s, 3H, 3-CH_3_); ^13^C NMR (100 MHz, DMSO-*d*_6_): δ 188.35 (C-8), 182.04 (C-5), 161.05 (C-6), 154.53 (C-10), 153.90 (C-9), 152.64 (C=O), 135.93 (C-9a), 134.16 (C-4a), 111.60 (C-10a), 110.10 (C-7), 109.56 (C-8a), 82.59 (C-3), 81.25 (C-2), 59.75 (C-1), 57.11 (6-OMe), 29.89 (C-4), 27.21 (3-CH_3_); EIMS *m/z* 362 [M]^+^ (46), 318 (17), 300 (100), 282 (29), 254 (19), 229 (25), 216 (10), 201 (11).

#### 3.3.5. General Procedure: Synthesis of Compounds **9–22**

To a solution of **1 **(1 equiv, 50 mg, 0.149 mmol) in 10 mL of methanol was added the corresponding amine (5 equiv). The reaction mixture was stirred at room temperature until the starting material disappeared (with aniline and 4-methoxyaniline, the reaction mixture was heated to 50 °C). The solvent was removed under reduced pressure. The resulting residue was purified on a silica gel column using dichloromethane–methanol as eluent, and then a C18 reversed phase silica gel column using methanol–water as eluent to obtain the corresponding products.

6-(Methylamino)-6-demethoxybostrycin (**9**). Yield 53.6%; red solid (MeOH); mp: 266–267 °C; IR (KBr): ν_max_ = 3532, 3457, 3389, 3319, 2973, 2928, 2843, 2808, 1584, 1517, 1418 cm^−1^; ^1^H NMR (400 MHz, DMSO-*d*_6_): δ 14.47 (s, 1H, 9-OH), 12.32 (s, 1H, 10-OH), 8.02 (q, *J *= 5.0 Hz, 1H, NH), 5.57 (s, 1H, 7-H), 5.14 (d, *J *= 4.8 Hz, 1H, 1-OH), 4.96 (d, *J *= 4.6 Hz, 1H, 2-OH), 4.75 (t, *J *= 4.8 Hz, 1H, 1-H), 4.50 (s, 1H, 3-OH), 3.52 (t, *J *= 4.6 Hz, 1H, 2-H), 2.83 (d, *J *= 5.0 Hz, 3H, NHCH_3_), 2.72 (d, *J *= 17.8 Hz, 1H, 4-H_b_), 2.64 (d, *J *= 17.8 Hz, 1H, 4-H_a_), 1.23 (s, 3H, 3-CH_3_); ^13^C NMR (125 MHz, DMSO-*d*_6_): δ 185.83 (C-8), 183.20 (C-5), 155.33 and 155.22 (C-9 and C-10), 150.44 (C-6), 139.49 (C-9a), 132.93 (C-4a), 109.60 (C-10a), 107.92 (C-8a), 98.58 (C-7), 76.38 (C-2), 69.36 (C-3), 68.56 (C-1), 34.75 (C-4), 29.08 (NHCH_3_), 25.68 (3-CH_3_); EIMS *m/z* 335 [M]^+^ (46), 317 (14), 288 (31), 261 (65), 246 (11), 233 (100), 218 (23), 204 (8).

6-(*n*-Propylamino)-6-demethoxybostrycin (**10**). Yield 50.2%; red solid (MeOH); mp: 210–211 °C; IR (KBr): ν_max_ = 3555, 3439, 3385, 3280, 3085, 2966, 2934, 2907, 2876, 1581, 1510, 1433 cm^−1^; ^1^H NMR (400 MHz, DMSO-*d*_6_): δ 14.51 (s, 1H, 9-OH), 12.33 (s, 1H, 10-OH), 7.97 (br t, 1H, NH), 5.67 (s, 1H, 7-H), 5.14 (d, *J *= 4.4 Hz, 1H, 1-OH), 4.95 (d, *J *= 4.2 Hz, 1H, 2-OH), 4.74 (t, *J *= 4.4 Hz, 1H, 1-H), 4.49 (s, 1H, 3-OH), 3.51 (t, *J *= 4.2 Hz, 1H, 2-H), 3.18 (q, *J *= 7.3 Hz, 2H, 1′-CH_2_), 2.72 (d, *J *= 17.8 Hz, 1H, 4-H_b_), 2.64 (d, *J *= 17.8 Hz, 1H, 4-H_a_), 1.60 (sextet, *J *= 7.4 Hz, 2H, 2′-CH_2_), 1.22 (s, 3H, 3-CH_3_), 0.91 (t, *J *= 7.5 Hz, 3H, 3′-CH_3_); ^13^C NMR (125 MHz, DMSO-*d*_6_): δ 185.87 (C-8), 183.30 (C-5), 155.27 and 155.25 (C-9 and C-10), 149.57 (C-6), 139.53 (C-9a), 132.89 (C-4a), 109.63 (C-10a), 107.83 (C-8a), 98.56 (C-7), 76.38 (C-2), 69.36 (C-3), 68.57 (C-1), 43.72 (C-1′), 34.74 (C-4), 25.67 (3-CH_3_), 20.67 (C-2′), 11.26 (C-3′); EIMS *m/z* 363 [M]^+^ (43), 345 (13), 316 (53), 302 (16), 289 (83), 261 (61), 232 (100), 218 (30).

6-(*n*-Butylamino)-6-demethoxybostrycin (**11**). Yield 48.5%; red solid (MeOH); mp: 236–237 °C; IR (KBr): ν_max_ = 3559, 3427, 3283, 3090, 2956, 2932, 2872, 1579, 1510, 1443 cm^–1^; ^1^H NMR (500 MHz, DMSO-*d*_6_): δ 14.47 (s, 1H, 9-OH), 12.32 (s, 1H, 10-OH), 7.89 (t, *J *= 5.4 Hz, 1H, NH), 5.65 (s, 1H, 7-H), 5.07 (d, *J *= 4.7 Hz, 1H, 1-OH), 4.88 (d, *J *= 4.8 Hz, 1H, 2-OH), 4.75 (t, *J *= 4.7 Hz, 1H, 1-H), 4.43 (s, 1H, 3-OH), 3.52 (t, *J *= 4.8 Hz, 1H, 2-H), 3.21 (dt, *J *= 5.4, 7.5 Hz, 2H, 1′-CH_2_), 2.73 (d, *J *= 17.8 Hz, 1H, 4-H_b_), 2.65 (d, *J *= 17.8 Hz, 1H, 4-H_a_), 1.57 (pentet, *J *= 7.5 Hz, 2H, 2′-CH_2_), 1.34 (sextet, *J *= 7.3 Hz, 2H, 3′-CH_2_), 1.23 (s, 3H, 3-CH_3_), 0.91 (t, *J *= 7.5 Hz, 3H, 4′-CH_3_); ^13^C NMR (125 MHz, DMSO-*d*_6_): δ 185.82 (C-8), 183.26 (C-5), 155.29 (C-9 and C-10), 149.53 (C-6), 139.52 (C-9a), 132.91 (C-4a), 109.62 (C-10a), 107.83 (C-8a), 98.50 (C-7), 76.38 (C-2), 69.36 (C-3), 68.58 (C-1), 41.80 (C-1′), 34.75 (C-4), 29.34 (C-2′), 25.67 (3-CH_3_), 19.60 (C-3′), 13.56 (C-4′); EIMS *m/z* 377 [M]^+^ (43), 359 (18), 341 (21), 303 (98), 275 (70), 260 (24), 232 (100), 218 (35).

6-(*n*-Hexylamino)-6-demethoxybostrycin (**12**). Yield 45%; red solid (MeOH); mp: 203–204 °C; IR (KBr): ν_max_ = 3557, 3384, 3285, 3089, 2955, 2929, 2859, 1578, 1512, 1436 cm^−1^; ^1^H NMR (400 MHz, DMSO-*d*_6_): δ 14.47 (s, 1H, 9-OH), 12.33 (s, 1H, 10-OH), 7.91 (t, *J *= 6.1 Hz, 1H, NH), 5.65 (s, 1H, 7-H), 5.08 (d, *J *= 4.8 Hz, 1H, 1-OH), 4.89 (d, *J *= 4.6 Hz, 1H, 3-OH), 4.75 (t, *J *= 4.8 Hz, 1H, 1-H), 4.44 (s, 1H, 3-OH), 3.52 (t, *J *= 4.6 Hz, 1H, 2-H), 3.21 (dt, *J *= 6.1, 7.0 Hz, 2H, 1′-CH_2_), 2.73 (d, *J *= 17.8 Hz, 1H, 4-H_b_), 2.65 (d, *J *= 17.8 Hz, 1H, 4-H_a_), 1.58 (pentet, *J *= 7.0 Hz, 2H, 2′-CH_2_), 1.37–1.26 (m, 6H, 3′, 4′ and 5′-CH_2_), 1.23 (s, 3H, 3-CH_3_), 0.87 (t, *J *= 7.1 Hz, 3H, 6′-CH_3_); ^13^C NMR (100 MHz, DMSO-*d*_6_): δ 185.90 (C-8), 183.36 (C-5), 155.31 and 155.27 (C-9 and C-10), 149.55 (C-6), 139.58 (C-9a), 132.92 (C-4a), 109.67 (C-10a), 107.87 (C-8a), 98.54 (C-7), 76.41 (C-2), 69.39 (C-3), 68.60 (C-1), 42.11 (C-1′), 34.77 (C-4), 30.87 (C-2′), 27.23 and 26.07 (C-3′ and C-4′), 25.70 (3-CH_3_), 21.97 (C-5′), 13.82 (C-6′); EIMS *m/z* 405 [M]^+^ (29), 387 (10), 358 (39), 331 (85), 303 (67), 382 (30), 323 (100), 218 (32).

6-(2′-Hydroxyethylamino)-6-demethoxybostrycin (**13**). Yield 30%; red solid (MeOH); mp: 202–203 °C; IR (KBr): ν_max_ = 3374, 2968, 2924, 2898, 2857, 1587, 1518, 1439, 1402 cm^–1^; ^1^H NMR (500 MHz, DMSO-*d*_6_): δ 14.45 (s, 1H, 9-OH), 12.31 (s, 1H, 10-OH), 7.70 (t, *J *= 6.0 Hz, 1H, NH), 5.73 (s, 1H, 7-H), 5.12 (d, *J *= 4.6 Hz, 1H, 1-OH), 4.92 (d, *J *= 4.4 Hz, 1H, 2-OH), 4.89 (t, *J *= 6.0 Hz, 1H, 2′-OH), 4.74 (t, *J *= 4.6 Hz, 1H, 1-H), 4.46 (s, 1H, 3-OH), 3.61 (q, *J *= 6.0 Hz, 2H, 2′-CH_2_), 3.51 (t, *J *= 4.4 Hz, 1H, 2-H), 3.29 (q, *J *= 6.0 Hz, 2H, 1′-CH_2_), 2.72 (d, *J *= 17.8 Hz, 1H, 4-H_b_), 2.65 (d, *J *= 17.8 Hz, 1H, 4-H_a_), 1.22 (s, 3H, 3-CH_3_); ^13^C NMR (125 MHz, DMSO-*d*_6_): δ 185.92 (C-8), 183.03 (C-5), 155.40 (C-9 and C-10), 149.79 (C-6), 139.56 (C-9a), 133.08 (C-4a), 109.55 (C-10a), 107.81 (C-8a), 98.95 (C-7), 76.38 (C-2), 69.36 (C-3), 68.58 (C-1), 58.41 (C-2′), 44.81 (C-1′), 34.78 (C-4), 25.68 (3-CH_3_); EIMS *m/z* 365 [M]^+^ (8), 349 (24), 327 (13), 314 (100), 298 (39), 282 (73), 276 (16), 232 (24).

6-[(2″-Hydroxyethoxy)ethylamino]-6-demethoxybostrycin (**14**). Yield 40.2%; red solid (MeOH); mp: 112–113 °C; IR (KBr): ν_max_ = 3376, 2973, 2922, 2872, 1584, 1516, 1439, 1373 cm^−1^; ^1^H NMR (400 MHz, DMSO-*d*_6_): δ 14.38 (s, 1H, 9-OH), 12.30 (s, 1H, 10-OH), 7.68 (t, *J *= 5.6 Hz, 1H, NH), 5.74 (s, 1H, 7-H), 5.14 (d, *J *= 4.8 Hz, 1H, 1-OH), 4.94 (d, *J *= 4.6 Hz, 1H, 2-OH), 4.75 (t, *J *= 4.8 Hz, 1H, 1-H), 4.68 (t, *J *= 5.2 Hz, 1H, 2″-OH), 4.50 (s, 1H, 3-OH), 3.65 (t, *J *= 5.6 Hz, 2H, 1″-CH_2_), 3.58–3.50 (m, 5H, 2-H, 2′-CH_2_ and 2″-CH_2_), 3.40 (q, *J *= 5.6 Hz, 2H, 1′-CH_2_), 2.74 (d, *J *= 17.8 Hz, 1H, 4-H_b_), 2.65 (d, *J *= 17.8 Hz, 1H, 4-H_a_), 1.24 (s, 3H, 3-CH_3_); ^13^C NMR (100 MHz, DMSO-*d*_6_): δ 186.59 (C-8), 183.63 (C-5), 155.87 and 155.84 (C-9 and C-10), 150.12 (C-6), 140.09 (C-9a), 133.61 (C-4a), 110.09 (C-10a), 108.34 (C-8a), 99.65 (C-7), 76.92 (C-2), 72.60 (C-2′), 69.96 (C-3), 69.12 (C-1), 67.93 (C-1″), 60.65 (C-2″), 42.65 (C-1′), 35.29 (C-4), 26.16 (3-CH_3_); EIMS *m/z* 409 [M]^+^ (2), 389 (24), 373 (4), 357 (5), 327 (9), 314 (100), 300 (27), 282 (31).

6-(Phenylamino)-6-demethoxybostrycin (**15**). Yield 60.4%; red solid (MeOH); mp: 226–227 °C; IR (KBr): ν_max_ = 3576, 3560, 3409, 3248, 3037, 2968, 2919, 2901, 2850, 1587, 1572, 1521, 1444 cm^−1^; ^1^H NMR (500 MHz, DMSO-*d*_6_): δ 14.16 (s, 1H, 9-OH), 12.47 (s, 1H, 10-OH), 9.50 (br s, 1H, NH), 7.51–7.28 (m, 5H, Ar-H), 6.07 (s, 1H, 7-H), 5.13 (br s, 1H, 1-OH), 4.91 (d, *J *= 4.5 Hz, 1H, 2-OH), 4.81 (br t, 1H, 1-H), 4.45 (s, 1H, 3-OH), 3.57 (t, *J *= 4.6 Hz, 1H, 2-H), 2.80 (d, *J *= 17.9 Hz, 1H, 4-H_b_), 2.72 (d, *J *= 17.9 Hz, 1H, 4-H_a_), 1.28 (s, 3H, 3-CH_3_); ^13^C NMR (125 MHz, DMSO-*d*_6_): δ 186.46 (C-8), 182.83 (C-5), 155.87 and 155.73 (C-9 and C-10), 147.33 (C-6), 139.49 (C-9a), 137.65 (C-1′), 133.97 (C-4a), 129.27 (C-3′ and C-5′), 125.63 (C-4′), 123.91 (C-2′ and C-6′), 109.63 (C-10a), 107.84 (C-8a), 101.33 (C-7), 76.40 (C-2), 69.36 (C-3), 68.51 (C-1), 34.86 (C-4), 25.68 (3-CH_3_); EIMS *m/z* 397 [M]^+^ (20), 377 (45), 361 (28), 345 (27), 324 (53), 295 (100), 280 (14), 266 (4).

6-(*p*-Methoxyphenylamino)-6-demethoxybostrycin (**16**). Yield 50.1%; red solid (MeOH); mp: 202–203 °C; IR (KBr): ν_max_ = 3379, 3260, 3032, 2964, 2921, 2838, 1585, 1522, 1440, 1418 cm^−1^; ^1^H NMR (400 MHz, DMSO-*d*_6_): δ 14.22 (s, 1H, 9-OH), 12.43 (s, 1H, 10-OH), 9.43 (br s, 1H, NH), 7.33 (d, *J *= 8.6 Hz, 2H, Ar-H), 7.05 (br d, *J *= 8.6 Hz, 2H, Ar-H), 5.88 (s, 1H, 7-H), 5.11 (br s, 1H, 1-OH), 4.91 (br s, 1H, 2-OH), 4.79 (br t, 1H, 1-H), 4.46 (s, 1H, 3-OH), 3.81 (s, 3H, 4′-OMe), 3.56 (br t, 1H, 2-H), 2.79 (d, *J *= 17.9 Hz, 1H, 4-H_b_), 2.70 (d, *J *= 17.9 Hz, 1H, 4-H_a_), 1.27 (s, 3H, 3-CH_3_); ^13^C NMR (100 MHz, DMSO-*d*_6_): δ 186.55 (C-8), 183.34 (C-5), 157.25 (C-4′), 155.58 and 155.51 (C-9 and C-10), 148.02 (C-6), 139.60 (C-9a), 133.67 (C-4a), 130.22 (C-1′), 125.83 (C-2′ and C-6′), 114.58 (C-3′ and C-5′), 109.75 (C-10a), 107.95 (C-8a), 100.50 (C-7), 76.47 (C-2), 69.45 (C-3), 68.58 (C-1), 55.34 (4′-OMe), 34.89 (C-4), 25.76 (3-CH_3_); EIMS *m/z* 427 [M]^+^ (29), 407 (74), 392 (53), 360 (44), 325 (41), 310 (33), 284 (18), 263 (100).

6-(Benzylamino)-6-demethoxybostrycin (**17**). Yield 52.6%; red solid (MeOH); mp: 153–154 °C; IR (KBr): ν_max_ = 3384, 3064, 2971, 2927, 2907, 1581, 1513, 1438, 1373 cm^−1^; ^1^H NMR (400 MHz, DMSO-*d*_6_): δ 14.30 (s, 1H, 9-OH), 12.36 (s, 1H, 10-OH), 8.51 (t, *J *= 6.4 Hz, 1H, NH), 7.36–7.25 (m, 5H, Ar-H), 5.58 (s, 1H, 7-H), 5.09 (d, *J *= 4.8 Hz, 1H, 1-OH), 4.90 (d, *J *= 4.6 Hz, 1H, 2-OH), 4.74 (t, *J *= 4.8 Hz, 1H, 1-H), 4.49 (d, *J *= 6.4 Hz, 2H, NHC*H*_2_Ph), 4.44 (s, 1H, 3-OH), 3.52 (t, *J *= 4.6 Hz, 1H, 2-H), 2.74 (d, *J *= 17.9 Hz, 1H, 4-H_b_), 2.66 (d, *J *= 17.9 Hz, 1H, 4-H_a_), 1.23 (s, 3H, 3-CH_3_); ^13^C NMR (100 MHz, DMSO-*d*_6_): δ 185.98 (C-8), 183.29 (C-5), 155.46 and 155.38 (C-9 and C-10), 149.54 (C-6), 139.57 (C-9a), 137.08 (C-1′), 133.24 (C-4a), 128.49 (C-3′ and C-5′), 127.16 and 127.12 (C-2′, C-4′ and C-6′), 109.67 (C-10a), 107.79 (C-8a), 99.84 (C-7), 76.41 (C-2), 69.38 (C-3), 68.56 (C-1), 45.25 (NHCH_2_), 34.79 (C-4), 25.70 (3-CH_3_); EIMS *m/z* 411 [M]^+^ (4), 391 (10), 359 (12), 337 (13), 302 (23), 284 (11), 218 (19), 204 (5), 91 (100).

6-(*p*-Methoxybenzylamino)-6-demethoxybostrycin (**18**). Yield 55.3%; red solid (MeOH); mp: 149–150 °C; IR (KBr): ν_max_ = 3375, 3072, 2968, 2932, 2912, 2837, 1581, 1513, 1438, 1399 cm^−1^; ^1^H NMR (400 MHz, DMSO-*d*_6_): δ 14.33 (s, 1H, 9-OH), 12.35 (s, 1H, 10-OH), 8.47 (t, *J *= 6.4 Hz, 1H, NH), 7.32–6.88 (m, 4H, Ar-H), 5.59 (s, 1H, 7-H), 5.10 (d, *J *= 4.8 Hz, 1H, 1-OH), 4.91 (d, *J *= 4.6 Hz, 1H, 2-OH), 4.74 (t, *J *= 4.8 Hz, 1H, 1-H), 4.45 (s, 1H, 3-OH), 4.41 (d, *J *= 6.4 Hz, 2H, NHC*H*_2_Ph), 3.73 (s, 3H, 4′-OMe), 3.51 (t, *J *= 4.6 Hz, 1H, 2-H), 2.73 (d, *J *= 17.8 Hz, 1H, 4-H_b_), 2.65 (d, *J *= 17.8 Hz, 1H, 4-H_a_), 1.22 (s, 3H, 3-CH_3_); ^13^C NMR (100 MHz, DMSO-*d*_6_): δ 185.94 (C-8), 183.37 (C-5), 158.44 (C-4′), 155.45 and 155.35 (C-9 and C-10), 149.40 (C-6), 139.59 (C-9a), 133.20 (C-4a), 128.89 and 128.55 (C-1′, C-2′ and C-6′), 113.91 (C-3′ and C-5′), 109.66 (C-10a), 107.80 (C-8a), 99.74 (C-7), 76.42 (C-2), 69.40 (C-3), 68.58 (C-1), 55.02 (4′-OMe), 44.74 (NHCH_2_), 34.80 (C-4), 25.72 (3-CH_3_); EIMS *m/z* 441 [M]^+^ (2), 425 (3), 407 (2), 389 (1), 320 (3), 302 (2), 274 (1), 232 (4), 121 (100).

6-(*p*-Fluorobenzylamino)-6-demethoxybostrycin (**19**). Yield 43.2%; red solid (MeOH); mp: 147–148 °C; IR (KBr): ν_max_ = 3376, 3076, 2971, 2928, 2858, 1582, 1510, 1442, 1420 cm^–1^; ^1^H NMR (400 MHz, DMSO-*d*_6_): δ 14.29 (s, 1H, 9-OH), 12.35 (s, 1H, 10-OH), 8.49 (t, *J *= 6.4 Hz, 1H, NH), 7.45–7.14 (m, 4H, Ar-H), 5.60 (s, 1H, 7-H), 5.09 (d, *J *= 4.7 Hz, 1H, 1-OH), 4.90 (d, *J *= 4.8 Hz, 1H, 2-OH), 4.74 (t, *J *= 4.7 Hz, 1H, 1-H), 4.47 (d, *J *= 6.4 Hz, 2H, NHC*H*_2_Ph), 4.44 (s, 1H, 3-OH), 3.52 (t, *J *= 4.8 Hz, 1H, 2-H), 2.74 (d, *J *= 17.8 Hz, 1H, 4-H_b_), 2.65 (d, *J *= 17.8 Hz, 1H, 4-H_a_), 1.23 (s, 3H, 3-CH_3_); ^13^C NMR (100 MHz, DMSO-*d*_6_): δ 186.02 (C-8), 183.26 (C-5), 161.31 (C-4′), 155.46 and 155.36 (C-9 and C-10), 149.42 (C-6), 139.55 (C-9a), 133.26 (C-4a), 129.25 and 129.17 (C-1′, C-2′ and C-6′), 115.21 (C-3′ and C-5′), 109.66 (C-10a), 107.78 (C-8a), 99.84 (C-7), 76.41 (C-2), 69.38 (C-3), 68.56 (C-1), 44.48 (NHCH_2_), 34.78 (C-4), 25.69 (3-CH_3_); ESIMS *m/z* 428 [M − 1]^−^.

6-(Piperidin-1-yl)-6-demethoxybostrycin (**20**). Yield 55%; red solid (MeOH); mp: 199–200 °C; IR (KBr): ν_max_ = 3557, 3399, 3054, 2928, 2855, 1597, 1576, 1548, 1447, 1429 cm^−1^; ^1^H NMR (400 MHz, DMSO-*d*_6_): δ 14.10 (s, 1H, 9-OH), 12.58 (s, 1H, 10-OH), 6.03 (s, 1H, 7-H), 5.08 (d, *J *= 4.9 Hz, 1H, 1-OH), 4.88 (d, *J *= 4.7 Hz, 1H, 2-OH), 4.78 (t, *J *= 4.9 Hz, 1H, 1-H), 4.42 (s, 1H, 3-OH), 3.58 (br s, 4H, 2′-CH_2_ and 6′-CH_2_), 3.55 (t, *J *= 4.7 Hz, 1H, 2-H), 2.76 (d, *J *= 17.9 Hz, 1H, 4-H_b_), 2.67 (d, *J *= 17.9 Hz, 1H, 4-H_a_), 1.68 (br s, 6H, 3′-CH_2_, 4′-CH_2_ and 5′-CH_2_), 1.25 (s, 3H, 3-CH_3_); ^13^C NMR (100 MHz, DMSO-*d*_6_): δ 185.33 (C-8), 184.27 (C-5), 156.12 and 155.91 (C-9 and C-10), 153.93 (C-6), 138.50 (C-9a), 134.27 (C-4a), 111.14 (C-10a), 109.30 (C-7), 108.02 (C-8a), 76.45 (C-2), 69.45 (C-3), 68.50 (C-1), 50.40 (C-2′ and C-6′), 35.00 (C-4), 25.75 (3-CH_3_), 25.46 (C-3′ and C-5′), 23.67 (C-4′); EIMS *m/z* 389 [M]^+^ (37), 371 (30), 353 (17), 316 (100), 300 (12), 286 (24), 258 (10), 242 (9).

6-(4′-Methylpiperidin-1-yl)-6-demethoxybostrycin (**21**). Yield 56.7%; red solid (MeOH); mp: 162–163 °C; IR (KBr): ν_max_ = 3559, 3408, 3055, 2948, 2927, 2855, 1597, 1580, 1550, 1451, 1432 cm^−1^; ^1^H NMR (400 MHz, DMSO-*d*_6_): δ 14.10 (s, 1H, 9-OH), 12.59 (s, 1H, 10-OH), 6.04 (s, 1H, 7-H), 5.09 (d, *J *= 4.8 Hz, 1H, 1-OH), 4.88 (d, *J *= 4.6 Hz, 1H, 2-OH), 4.78 (t, *J *= 4.8 Hz, 1H, 1-H), 4.42 (s, 1H, 3-OH), 4.13 (m, 2H, 2′-H_b_ and 6′-H_b_), 3.55 (t, *J *= 4.6 Hz, 1H, 2-H), 3.05 (m, 2H, 2′-H_a_ and 6′-H_a_), 2.76 (d, *J *= 17.9 Hz, 1H, 4-H_b_), 2.68 (d, *J *= 17.9 Hz, 1H, 4-H_a_), 1.71 (m, 1H, 4′-H), 1.70 (m, 2H, 3′-H_b_ and 5′-H_b_), 1.27 (m, 2H, 3′-H_a_ and 5′-H_a_), 1.26 (s, 3H, 3-CH_3_), 0.97 (d, *J *= 6.1 Hz, 3H, 4′-CH_3_); ^13^C NMR (100 MHz, DMSO-*d*_6_): δ 185.13 (C-8), 184.04 (C-5), 156.33 and 156.05 (C-9 and C-10), 153.83 (C-6), 138.48 (C-9a), 134.32 (C-4a), 111.11 (C-10a), 109.46 (C-7), 107.99 (C-8a), 76.43 (C-2), 69.42 (C-3), 68.50 (C-1), 49.63 and 49.56 (C-2′ and C-6′), 34.99 (C-4), 33.61 and 33.52 (C-3′ and C-5′), 29.99 (C-4′), 25.71 (3-CH_3_), 21.41 (4′-CH_3_); EIMS *m/z* 403 [M]^+^ (33), 385 (34), 367 (50), 330 (100), 314 (31), 300 (28), 286 (20), 270 (15).

6-(4′-Phenylpiperidin-1-yl)-6-demethoxybostrycin (**22**). Yield 45.6%; red solid (MeOH); mp: 221–222 °C; IR (KBr): ν_max_ = 3538, 3397, 3033, 2956, 2933, 2900, 2870, 1570, 1539, 1495, 1455 cm^−1^; ^1^H NMR (400 MHz, DMSO-*d*_6_): δ 14.07 (s, 1H, 9-OH), 12.60 (s, 1H, 10-OH), 7.35–7.26 (m, 4H, Ar-H), 7.21 (m, 1H, Ar-H), 6.10 (s, 1H, 7-H), 5.12 (d, *J *= 4.7 Hz, 1H, 1-OH), 4.90 (d, *J *= 4.8 Hz, 1H, 2-OH), 4.77 (t, *J *= 4.7 Hz, 1H, 1-H), 4.44 (s, 1H, 3-OH), 4.27 (m, 2H, 2′-H_b_ and 6′-H_b_), 3.53 (t, *J *= 4.8 Hz, 1H, 2-H), 3.15 (m, 2H, 2′-H_a_ and 6′-H_a_), 2.83 (m, 1H, 4′-H), 2.75 (d, *J *= 17.9 Hz, 1H, 4-H_b_), 2.66 (d, *J *= 17.9 Hz, 1H, 4-H_a_), 1.91–1.76 (m, 4H, 3′-CH_2_ and 5′-CH_2_), 1.24 (s, 3H, 3-CH_3_); ^13^C NMR (100 MHz, DMSO-*d*_6_): δ 185.24 (C-8), 184.04 (C-5), 156.35 and 156.12 (C-9 and C-10), 153.80 (C-6), 145.33 (C-1″), 138.53 (C-9a), 134.39 (C-4a), 128.38, 126.67 and 126.19 (C-2″, C-3″, C-4″, C-5″ and C-6″), 111.12 (C-10a), 109.84 (C-7), 108.01 (C-8a), 76.43 (C-2), 69.42 (C-3), 68.49 (C-1), 50.00 and 49.92 (C-2′ and C-6′), 41.32 (C-4′), 34.99 (C-4), 32.77 and 32.66 (C-3′ and C-5′), 25.71 (3-CH_3_); EIMS *m/z* 465 [M]^+^ (53), 445 (85), 429 (91), 413 (66), 392 (100), 376 (12), 362 (16), 328 (62).

#### 3.3.6. General Procedure: Synthesis of Compounds **23–29**

To a solution of **1** (1 equiv, 50 mg, 0.149 mmol) and triethylamine (8 equiv) in 10 mL of methanol was added the corresponding thiol (4 equiv). The reaction mixture was stirred at 0–5 °C until the starting material disappeared. The solvent was removed under reduced pressure. The resulting residue was purified on a silica gel column using dichloromethane–methanol as eluent, and then a C18 reversed phase silica gel column using methanol–water as eluent to obtain the corresponding products.

6,7-bis(Ethylthio)-6-demethoxybostrycin (**23**). Yield 60.4%; red solid (MeOH); mp: 180–181 °C; IR (KBr): ν_max_ = 3561, 3379, 2968, 2923, 2865, 1604, 1572, 1486, 1447, 1426 cm^−1^; ^1^H NMR (400 MHz, DMSO-*d*_6_): δ 13.22 (s, 1H, 9-OH), 13.04 (s, 1H, 10-OH), 5.29 (d, *J *= 4.5 Hz, 1H, 1-OH), 4.94 (d, *J *= 4.4 Hz, 1H, 2-OH), 4.75 (br t, 1H, 1-H), 4.49 (s, 1H, 3-OH), 3.53 (t, *J *= 4.4 Hz, 1H, 2-H), 3.30–3.24 (m, 4H, 1′-CH_2_ and 1″-CH_2_), 2.74 (d, *J *= 18.5 Hz, 1H, 4-H_b_), 2.67 (d, *J *= 18.5 Hz, 1H, 4-H_a_), 1.24–1.20 (m, 9H, 3-CH_3_, 2′-CH_3_ and 2″-CH_3_); ^13^C NMR(100 MHz, DMSO-*d*_6_): δ 175.67 (C-5), 175.55 (C-8), 164.61 (C-9), 163.16 (C-10), 145.75 (C-7), 145.07 (C-6), 138.58 and 138.54 (C-4a and C-9a), 109.69 (C-10a), 109.32 (C-8a), 76.36 (C-2), 69.24 (C-3), 68.17 (C-1), 34.95 (C-4), 28.70 and 28.64 (C-1′ and C-1″), 25.53 (3-CH_3_), 15.03 and 15.00 (C-2′ and C-2″); EIMS *m/z* 426 [M]^+^ (8), 408 (10), 390 (3), 379 (21), 361 (100), 345 (28), 333 (79), 319 (22).

6,7-bis(*n*-Butylthio)-6-demethoxybostrycin (**24**). Yield 51.2%; red solid (MeOH); mp: 124–125 °C; IR (KBr): ν_max_ = 3551, 3400, 2958, 2929, 2871, 1599, 1532, 1431, 1377 cm^−1^; ^1^H NMR (400 MHz, DMSO-*d*_6_): δ 13.23 (s, 1H, 9-OH), 13.05 (s, 1H, 10-OH), 5.29 (br s, 1H, 1-OH), 4.94 (br s, 1H, 2-OH), 4.75 (br s, 1H, 1-H), 4.49 (s, 1H, 3-OH), 3.53 (d, *J *= 4.4 Hz, 1H, 2-H), 3.29–3.20 (m, 4H, 1′-CH_2_ and 1″-CH_2_), 2.73 (d, *J *= 18.4 Hz, 1H, 4-H_b_), 2.67 (d, *J *= 18.4 Hz, 1H, 4-H_a_), 1.58–1.46 (m, 4H, 2′-CH_2_ and 2″-CH_2_), 1.39 (sextet, *J *= 7.2 Hz, 4H, 3′-CH_2_ and 3″-CH_2_), 1.23 (s, 3H, 3-CH_3_), 0.86 (br t, *J *= 7.2 Hz, 6H, 4′-CH_3_ and 4″-CH_3_); ^13^C NMR (100 MHz, DMSO-*d*_6_): δ 175.85 (C-5), 175.74 (C-8), 164.55 (C-9), 163.07 (C-10), 146.16 (C-7), 145.52 (C-6), 138.59 and 138.55 (C-4a and C-9a), 109.76 (C-10a), 109.37 (C-8a), 76.39 (C-2), 69.30 (C-3), 68.20 (C-1), 35.00 (C-4), 34.15 and 34.09 (C-1′ and C-1″), 31.84 and 31.81 (C-2′ and C-2″), 25.61 (3-CH_3_), 21.10 (C-3′ and C-3″), 13.37 (C-4′ and C-4″); EIMS *m/z* 482 [M]^+^ (2), 464 (3), 446 (8), 430 (2), 405 (28), 389 (100), 373 (31), 361 (14).

6,7-bis(*n*-Hexylthio)-6-demethoxybostrycin (**25**). Yield 42.6%; red solid (MeOH); mp: 112–113°C; IR (KBr): ν_max_ = 3545, 3370, 2956, 2925, 2854, 1598, 1433, 1419, 1379 cm^−1^; ^1^H NMR (400 MHz, DMSO-*d*_6_): δ 13.23 (s, 1H, 9-OH), 13.05 (s, 1H, 10-OH), 5.29 (d, *J *= 4.9 Hz, 1H, 1-OH), 4.94 (br s, 1H, 2-OH), 4.75 (br t, 1H, 1-H), 4.49 (s, 1H, 3-OH), 3.53 (br d, 1H, 2-H), 3.29–3.19 (m, 4H, 1′-CH_2_ and 1″-CH_2_), 2.73 (d, *J *= 18.4 Hz, 1H, 4-H_b_), 2.67 (d, *J *= 18.4 Hz, 1H, 4-H_a_), 1.59–1.47 (m, 4H, 2′-CH_2_ and 2″-CH_2_), 1.44–1.30 (m, 4H, 3′-CH_2_ and 3″-CH_2_), 1.23 (br s, 11H, 3-CH_3_, 4′-CH_2_, 4″-CH_2_, 5′-CH_2_ and 5″-CH_2_), 0.87–0.79 (m, 6H, 6′-CH_3_ and 6″-CH_3_); ^13^C NMR (100 MHz, DMSO-*d*_6_): δ 175.87 (C-5), 175.75 (C-8), 164.52 (C-9), 163.03 (C-10), 146.17 (C-7), 145.53 (C-6), 138.58 and 138.54 (C-4a and C-9a), 109.74 (C-10a), 109.36 (C-8a), 76.38 (C-2), 69.30 (C-3), 68.20 (C-1), 34.99 (C-4), 34.48 and 34.41 (C-1′ and C-1″), 30.65, 29.71, 29.68 and 27.58 (C-2′, C-2″, C-3′, C-3″, C-4′ and C-4″), 25.60 (3-CH_3_), 21.91 (C-5′ and C-5″), 13.76 (C-6′ and C-6″); EIMS *m/z* 538 [M]^+^ (4), 502 (4), 433 (26), 417 (100), 401 (21), 389 (12), 347 (7), 335 (20).

6,7-bis(2′-Hydroxyethylthio)-6-demethoxybostrycin (**26**). Yield 28.7%; red solid (MeOH); mp: 156–158 °C; IR (KBr): ν_max_ = 3560, 3405, 2960, 2928, 2874, 2862, 1606, 1573, 1488, 1426 cm^−1^; ^1^H NMR (400 MHz, DMSO-*d*_6_): δ 13.17 (s, 1H, 9-OH), 12.99 (s, 1H, 10-OH), 5.27 (d, *J *= 4.3 Hz, 1H, 1-OH), 4.92 (br d, 1H, 2-OH), 4.89 and 4.88 (each br t, 1H, 2′-OH and 2″-OH), 4.77 (br t, 1H, 1-H), 4.47 (s, 1H, 3-OH), 3.61 (q, *J *= 4.8 Hz, 4H, 2′-CH_2_ and 2″-CH_2_), 3.55 (br t, 1H, 2-H), 3.41–3.32 (m, 4H, 1′-CH_2_ and 1″-CH_2_), 2.76 (d, *J *= 18.4 Hz, 1H, 4-H_b_), 2.69 (d, *J *= 18.4 Hz, 1H, 4-H_a_), 1.25 (s, 3H, 3-CH_3_); ^13^C NMR (100 MHz, DMSO-*d*_6_): δ 177.50 (C-5), 177.37 (C-8), 162.54 (C-9), 160.90 (C-10), 146.93 (C-7), 146.41 (C-6), 138.10 and 138.02 (C-4a and C-9a), 109.91 (C-10a), 109.49 (C-8a), 76.45 (C-2), 69.36 (C-3), 68.26 (C-1), 61.05 (C-2′ and C-2″), 37.13 and 37.09 (C-1′ and C-1″), 35.06 (C-4), 25.68 (3-CH_3_); ESIMS *m/z* 457 [M − 1]^−^.

6,7-bis(3′-Hydroxy-*n*-propylthio)-6-demethoxybostrycin (**27**). Yield 32.5%; red solid (MeOH); mp: 150–151 °C; IR (KBr): ν_max_ = 3550, 3423, 2993, 2925, 2874, 2855, 1599, 1528, 1430, 1403, 1375 cm^−1^; ^1^H NMR (400 MHz, DMSO-*d*_6_): δ 13.20 (s, 1H, 9-OH), 13.03 (s, 1H, 10-OH), 5.25 (d, *J *= 4.3 Hz, 1H, 1-OH), 4.90 (d, *J *= 4.2 Hz, 1H, 2-OH), 4.75 (br t, 1H, 1-H), 4.50–4.43 (m, 3H, 3′-OH, 3″-OH and 3-OH), 3.52 (t, *J *= 4.2 Hz, 1H, 2-H), 3.47 (q, *J *= 6.4 Hz, 4H, 3′-CH_2_ and 3″-CH_2_), 3.36–3.28 (m, 4H, 1′-CH_2_ and 1″-CH_2_), 2.74 (d, *J *= 18.4 Hz, 1H, 4-H_b_), 2.67 (d, *J *= 18.4 Hz, 1H, 4-H_a_), 1.68 (pentet, *J *= 6.4 Hz, 4H, 2′-CH_2_ and 2″-CH_2_), 1.23 (s, 3H, 3-CH_3_); ^13^C NMR (100 MHz, DMSO-*d*_6_): δ 176.23 (C-5), 176.12 (C-8), 164.14 (C-9), 162.61 (C-10), 146.32 (C-7), 145.73 (C-6), 138.51 and 138.48 (C-4a and C-9a), 109.85 (C-10a), 109.45 (C-8a), 76.43 (C-2), 69.35 (C-3), 68.23 (C-1), 59.09 (C-3′ and C-3″), 35.04 (C-4), 33.07 and 33.04 (C-1′ and C-1″), 31.46 and 31.40 (C-2′ and C-2″), 25.67 (3-CH_3_); ESIMS *m/z* 485 [M − 1]^−^.

6,7-(Ethan-1′,2′-yl-dithio)-6-demethoxybostrycin (**28**). Yield 24.8%; red solid (MeOH); mp: >300 °C; IR (KBr): ν_max_ = 3417, 2966, 2925, 2857, 1588, 1513, 1436, 1375 cm^−1^; ^1^H NMR (400 MHz, DMSO-*d*_6_): δ 12.84 (s, 1H, 9-OH), 12.67 (s, 1H, 10-OH), 5.26 (br s, 1H, 1-OH), 4.94 (d, *J *= 4.4 Hz, 1H, 2-OH), 4.75 (d, *J *= 4.6 Hz, 1H, 1-H), 4.49 (s, 1H, 3-OH), 3.53 (t, *J *= 4.4 Hz, 1H, 2-H), 3.35 (br s, 4H, 1′-CH_2_ and 2′-CH_2_), 2.75 (d, *J *= 18.2 Hz, 1H, 4-H_b_), 2.68 (d, *J *= 18.2 Hz, 1H, 4-H_a_), 1.23 (s, 3H, 3-CH_3_); ^13^C NMR (100 MHz, DMSO-*d*_6_): δ 180.54 and 180.42 (C-5 and C-8), 157.65 (C-9), 155.72 (C-10), 140.74 (C-7), 140.20 (C-6), 137.39 and 137.23 (C-4a and C-9a), 108.17 (C-10a), 107.66 (C-8a), 76.43 (C-2), 69.29 (C-3), 68.30 (C-1), 35.11 (C-4), 26.17 and 26.08 (C-1′ and C-2′), 25.67 (3-CH_3_); EIMS *m/z* 396 [M]^+^ (6), 376 (28), 360 (100), 344 (47), 332 (22), 307 (23), 294 (13), 279 (12).

6,7-(Butan-2′,3′-yl-dithio)-6-demethoxybostrycin (**29**). Yield 63.5%; red solid (MeOH); mp: 146–147 °C; IR (KBr): ν_max_ = 3446, 3250, 2960, 2916, 2859, 1589, 1516, 1441, 1418 cm^−1^; ^1^H NMR (400 MHz, DMSO-*d*_6_): δ 12.85 (s, 1H, 9-OH), 12.69 (s, 1H, 10-OH), 5.22 (br s, 1H, 1-OH), 4.91 (d, *J *= 4.6 Hz, 1H, 2-OH), 4.75 (d, *J *= 4.5 Hz, 1H, 1-H), 4.45 (s, 1H, 3-OH), 3.67 and 3.48 (each m, 1H, 2′-CH and 3′-CH), 3.54 (t, *J *= 4.6 Hz, 1H, 2-H), 2.75 (d, *J *= 18.3 Hz, 1H, 4-H_b_), 2.68 (d, *J *= 18.3 Hz, 1H, 4-H_a_), 1.32 and 1.30 (each d, *J *= 6.2 Hz, 3H, 1′-CH_3_ and 4′-CH_3_), 1.23 (s, 3H, 3-CH_3_); ^13^C NMR (100 MHz, DMSO-*d*_6_): δ 180.69 and 180.66 (C-5 and C-8), 157.58 (C-9), 155.64 (C-10), 139.83 (C-7), 138.71 (C-6), 137.39 and 137.18 (C-4a and C-9a), 108.38 (C-10a), 107.86 (C-8a), 76.46 (C-2), 69.33 (C-3), 68.32 (C-1), 35.11 (C-4), 25.70 (3-CH_3_), 22.99 and 22.89 (C-2′ and C-3′), 17.60 (C-1′ and C-4′); EIMS *m/z* 424 [M]^+^ (8), 406 (5), 388 (3), 351 (29), 335 (5), 322 (9), 307 (5), 295 (13), 71 (100).

### 3.4. Antitumor Activity *in Vitro*

#### 3.4.1. Cell Culture

MCF-7, MDA-MB-435, A549, HepG2 and HCT-116 cells were cultured in Dulbecco’s modification Eagle’s medium (DMEM, Invitrogen, Carlsbad, CA, USA) supplemented with 10% fetal bovine serum (FBS, Hyclone, Logan, UT, USA), 2 mM L-glutamine, 100 μg/mL streptomycin and 100 U/mL penicillin (Invitrogen). MCF-10A cells were cultured in keratinocyte serum free medium (KSFM) supplemented with 0.1–0.2 ng/mL human recombinant epidermal growth factor and 20–30 μg/mL bovine pituitary extract (Invitrogen). The cells were incubated at 37 °C in a humidified atmosphere with 5% CO_2_.

#### 3.4.2. Assessment of Antitumor Activity by MTT Assay

Cells were harvested during logarithmic growth phase and seeded in 96-well plates at a density of 1 × 10^4^ cells/mL, and cultured at 37 °C in a humidified incubator (5% CO_2_) for 24 h, followed by exposure to various concentrations of compounds tested for 48 h. Subsequently 20 μL of MTT (Genview, Houston, TX, USA) solution (5 mg/mL) was added to each well and mixed, the cells were then incubated for an additional 4 h. Culture supernatant was moved, 150 μL of DMSO (Sangon Biotech, Shanghai, China) was added to each well to fully dissolve the MTT-formazan crystals. Cell growth inhibition was determined by measuring the absorbance (Abs) at λ = 570 nm using a microplate reader and calculated according to the following equation: 

   Growth inhibition = (1 − OD of treated cells/OD of control cells) × 100% 

The half maximal inhibitory concentrations (IC_50_) were obtained from liner regression analysis of the concentration-response curves plotted for each tested compound.

## 4. Conclusions

This paper has reported the synthesis and biological evaluation of a novel class of bostrycin derivatives against a panel of tumor cells along with one type of immortalized human breast epithelial cells. Encouragingly, some modified compounds displayed superior cytotoxicity over the parent compound bostrycin and equal anticancer potency to epirubicin which served as a reference anticancer drug. However, the majority of compounds also exhibited marked cytotoxicity towards MCF-10A cells. Preliminary SAR study indicated that the enhanced cytotoxicity was fulfilled by dioxylcarbonyl groups at C-2 and C-3 positions, tertiary amino groups at C-6 position and alkylthio groups at C-6 and C-7 positions of the bostrycin. These positive results could serve as a valuable guideline for further research on the structural optimization, mechanism study and development of bostrycin derivatives as novel antitumor agents.
